# Isolation and culture of primary adult skin fibroblasts from the Asian elephant (*Elephas maximus*)

**DOI:** 10.7717/peerj.4302

**Published:** 2018-01-24

**Authors:** Puntita Siengdee, Sarisa Klinhom, Chatchote Thitaram, Korakot Nganvongpanit

**Affiliations:** 1 Animal Bone and Joint Research Laboratory, Department of Veterinary Biosciences and Public Health, Faculty of Veterinary Medicine, Chiang Mai University, Chiang Mai, Thailand; 2 Center of Excellence in Elephant and Wildlife Research, Faculty of Veterinary Medicine, Chiang Mai University, Chiang Mai, Thailand; 3 Excellence Center in Veterinary Bioscience, Department of Veterinary Biosciences and Public Health, Faculty of Veterinary Medicine, Chiang Mai University, Chiang Mai, Thailand

**Keywords:** Asian elephant, Culture, Fibroblasts, Skin

## Abstract

**Background:**

Primary cultures from Asian elephants (*Elephas maximus*) allow scientists to obtain representative cells that have conserved most of their original characteristics, function, physiology and biochemistry. This technique has thus gained significant importance as a foundation for further cellular, cell biology and molecular research. Therefore, the aim of this study was to describe conditions for the successful establishment of primary adult fibroblasts from Asian elephant carcasses.

**Methods:**

Ear tissue sample collection from Asian elephant carcasses and our recommendations are given. We describe here a simple modified protocol for successful isolation and maintenance of primary adult fibroblasts from elephant ear skin. Ear samples from each individual (five 3 × 3 cm^2^ pieces) were brought to the laboratory within 3 h after collection, kept in transportation medium at 0–4 °C. The ear tissues were prepared by a combination of 10% collagenase type II digestion procedure together with a simple explant procedure. Primary fibroblasts were cultured at 37 °C in Dulbecco’s modified Eagle’s medium (DMEM) with 20% fetal calf serum (FCS) in a humidified atmosphere containing 5% CO_2_. After the third passage, fibroblasts were routinely trypsinized with 0.25% trypsin/EDTA and cultured in DMEM with 10% FCS at 37 °C and 5% CO_2_. Traditional cell counting method was used to measure cell viability and growth curve. Long-term storage of cells used freezing medium consisting of 40% FCS (v/v).

**Results:**

We explored the most suitable conditions during sample collection (post-mortem storage time and sample storage temperature), which is the most important step in determining primary outgrowth. Our study successfully established and cultured primary adult skin fibroblasts obtained from post-mortem *E. maximus* ear skin tissues from six carcasses, with a success rate of around 83.3%. Outgrowth could be seen 4–12 days after explantation, and epithelial-like cells were found after 4–7 days of culture, while fibroblasts appeared at around day 7–10. The fibroblasts had viability and post-freezing recovery rates of around 97.3 ± 4.3% and 95.5 ± 7.3%, respectively, and doubling time was about 25 h (passage 6).

**Discussion:**

To our knowledge, this report is the first to describe primary cell cultures derived from adult Asian elephant skin. Future studies should benefit from the information and useful suggestions herein, which may be used as a standard method for establishing primary skin fibroblast cultures in future experiments.

## Introduction

The elephant is the largest land mammal and is the only living species of the genus *Loxodonta* and *Elephas.* The Asian elephant (*Elephas maximus*) distributed in Asia, including mainland Asia and the islands of Sri Lanka, Borneo and Sumatra (Shoshani & Eisenberg, 1982). Four subspecies have been described: *Elephas maximus maximus* (Sri Lanka); *Elephas maximus indicus* (mainland Asia, including India, Nepal, Bangladesh, Bhutan, Myanmar, Thailand, Malay Peninsula, Laos, China, Cambodia and Vietnam); *Elephas maximus sumatranus* (Sumatra) (Sukumar, 1992); and a new subspecies (based on mitochondrial DNA), *Elephas maximus borneensis* (Fernando et al., 2003) called the Borneo pygmy elephant, from the island of Borneo (specifically the Malaysian states of Sabah and Kalimantan).

Most domesticated elephants today are found working in the tourism industry, particularly throughout Thailand. However, populations of domesticated elephants have declined more than 97% over the past century (Magda et al., 2015). Elephants are listed as protected animals under Thailand’s *Wild Animal Reservation* and Protection *Act*, B.E. 2535 (1992). In recent decades, numerous organizations have been established to provide better lives for the elephants, including improved healthcare and protection from exploitation by the tourism and logging industries. However, health issues still exist, with the major problem being wounds and abscesses (Angkawanish et al., 2009). Damaged skin consequently causes painful lesions on the elephant’s body. A high prevalence (∼64.4%) of active lesions, most located on the back region, was found to be associated with working conditions (Magda et al., 2015), and causing trauma and death in elephants at a rate of around 2.2% of major postmortem pathologic findings reported by survey respondents (Miller et al., 2015).

At the same time, wild elephants are suffering from poaching and severe habitat loss via encroachment and deforestation. Destruction of the forests gives rise to serious conflicts with people that share the same habitat, usually ending with more elephants being poisoned, injured or even killed. Effective management of the environment and good medical treatment are required in order to resolve these issues and enhance the animals’ well-being. However, there is little published literature on the treatment of elephant diseases and injuries, and a lack of basic information on Asian elephants from the cellular to the organismal level.

Cell culture technology has become a widely used method in biology, medical research and applications. Establishing primary cultures of fibroblasts allows researchers to obtain representative cells that have conserved most of their original characteristics and functions, which is an important foundation for further cell biology and cell engineering. Cryopreservation of animal cells is an excellent technique for long-term preservation of animal genetic resources, which is critical to guarantee genomics and genetic analyses (Groeneveld et al., 2008; Guan et al., 2010; Mestre-Citrinovitz et al., 2016). Only two elephant cell lines are available in the cell bank at the present time: LACF-NaNaI (RIKEN Cell Bank: RCB2319) and LACF-NaNaII (RIKEN Cell Bank: RCB2320), which were derived from the gum and ear, respectively, of the African savannah elephant.

To our knowledge, two previous studies briefly mentioned successful methods of isolating skin fibroblasts from Asian elephant skin, by direct culture of elephant skin explants (Sathanawongs et al., 2010) and trypsin digestion procedure (Techakumphu et al., 2017). However in these procedures, fibroblasts were limited to those isolated from young or stillborn donors with thin connective tissues that provided fast-growing fibroblasts and generally possessed greater proliferative capacity and good characteristics and function (Brink, Bernstein & Nicoll, 2009; Sriram, Bigliardi & Bigliardi-Qi, 2015). Even though dense tissues from older donors are less desirable for fibroblast culture establishment, adult elephant carcasses are more readily available and hence more frequently obtained. Isolating adult skin fibroblasts from direct culture of elephant skin explants (Aoued & Singh, 2015; Singh & Ma, 2014; Vangipuram et al., 2013) and also with collagenase digestion technique (Seluanov, Vaidya & Gorbunova, 2010) was ineffective in our preliminary study.

Therefore, the present study aimed to establish primary elephant skin fibroblasts taken from post-mortem adult Asian elephant ear skin tissues. Moreover, we have explored preliminary information about the development of methods of approach, problems and recommendations, and useful suggestions for culturing primary skin fibroblasts from an adult Asian elephant, to serve as standard methods for regular establishment of primary skin fibroblast cultures, which is of significant importance for further biological studies, including cell biology and cellular and molecular research.

## Materials and Methods

### Tissue sample collection

Tissue samples were obtained from six Asian elephant carcasses housed at different facilities/field settings (Licence number U1006312558). Details of the elephants are given in Table 1, including age, sex, cause of death, location, and length of time after death until samples were brought to the laboratory.

**Table 1:** Details of the elephant carcasses used in this study.

Animal	Age (years)	Sex	Cause of death	Location	Hours post-mortem
E1	40	Female	The necropsy indicated death from endotoxic shock due to impaction colic	Hang Chat district, Lampang, Thailand	6 h (including 1.5 h in transportation medium)
E2	33	Female	Found dead in enclosure; a necropsy was not performed	Mae Taeng district, Chiang Mai, Thailand	9 h (including 3 h in transportation medium)
E3	68	Male	Complications resulting from large bowel obstruction (food)	Mae Rim district, Chiang Mai, Thailand	7 h (including 2.5 h in transportation medium)
E4	∼35–40	Female	The necropsy revealed that death was due to bleeding in the abdomen	Mae Taeng district, Chiang Mai, Thailand	8 h (including 2 h in transportation medium)
E5	30	Female	Signs of ataxia for a month before death related to suspected nervous system disorder	Mae Taeng district, Chiang Mai, Thailand	8 h (including 2 h in transportation medium)
E6	2.9	Female	The necropsy indicated death from endotoxic shock as a result of *Clostridium* species infection	Mae Wang district, Chiang Mai, Thailand	20 h (including 1 h in transportation medium) (*No outgrowth*)

Reagents used in this study are listed in Table 2, including the main components of reagents and medium formulations and their vendor and concentration.

**Table 2:** Components of reagent and medium formulations.

Name	Components	Vendor and stock concentration
Routine antibiotic dose	1× antibiotic/antimycotic (containing 100 units/mL of streptomycin, 100 units/mL of penicillin and 0.25 µg/mL of amphotericin B)	• Antibiotic/antimycotic (100X) (Gibco™; Thermo Fisher Scientific, Waltham, MA, USA) stock concentration contains 10,000 units/mL of penicillin, 10,000 µg/mL of streptomycin, and 25 µg/mL of amphotericin B • Dulbecco’s modified Eagle’s medium; DMEM (Gibco; Thermo Fisher Scientific) • Phosphate-buffered saline (PBS) (10×) (Gibco; Thermo Fisher Scientific) • Gentamicin (50 mg/mL) (Gibco; Thermo Fisher Scientific) • Collagenase type II (Sigma-Aldrich, St. Louis, Mo, USA) stock concentration contains 1 mg/mL of collagenase II in DMEM w/o serum • Heat-inactivated fetal calf serum (FCS) (PAA Laboratories, Pasching, Austria) • Trypsin-EDTA (0.5%), no phenol red (Gibco; Thermo Fisher Scientific) • DMSO (Sigma-Aldrich)
**Tissue sample collection**			
Povidone–iodine solution	10% w/v povidone in 70% v/v isopropyl alcohol	
Chlorhexidine solution	4% chlorhexidine gluconate w/v in purified water	
Transportation medium	DMEM w/o serum + 10× antibiotic/antimycotic	
**Explant preparation**			
Washing PBS	PBS + 10× antibiotic/antimycotic + 50 µg/mL gentamicin	
Washing DMEM	DMEM w/o serum + 10× antibiotic/antimycotic + 50 µg/mL gentamicin	
Digestion medium	DMEM + 10% collagenase type II (v/v) + 10× antibiotic/antimycotic	
Explant medium	DMEM + 20% FCS + 1× antibiotic/antimycotic	
**Establishing secondary cultures**			
0.1% trypsin/EDTA	PBS + 0.1% trypsin/EDTA	
Growth medium	DMEM + 10% FCS + 1× antibiotic/antimycotic	
**Routine trypsinization, cryopreservation and resuscitation of fibroblasts**			
0.25% trypsin/EDTA	PBS + 0.25% trypsin/EDTA	
Freezing medium	50% DMEM + 10% DMSO + 40% FCS (v/v)	

Ear samples were excised after the mahout had conducted a death ritual or funeral ceremony according to Thai belief (Keyes, 1977), which occurred around 2 to 14 h after death. Ear skins were washed with water and scrubbed with povidone–iodine or chlorhexidine solution, using an impregnated brush to remove any remaining dirt, then rinsed with phosphate-buffered saline (PBS) or normal saline and dried with a sterile gauze pad. Ear samples were cut with scissors into 3 × 3 cm^2^ pieces; these were transferred to a sterile 50 mL test tube containing 25 mL of transportation medium with ten times the normal concentration of a routine antibiotic dose of antibiotic/antimycotic. Note that the solution volume should be enough to entirely cover the ear samples. The collection tube with tissue samples was then transported to the laboratory in a chilled carrier at 0−4 °C (on ice or in the presence of ice packs, but not in direct contact with the coolant to avoid sample freezing) within 3 h after collection.

### Explant preparation

**Figure 1:**
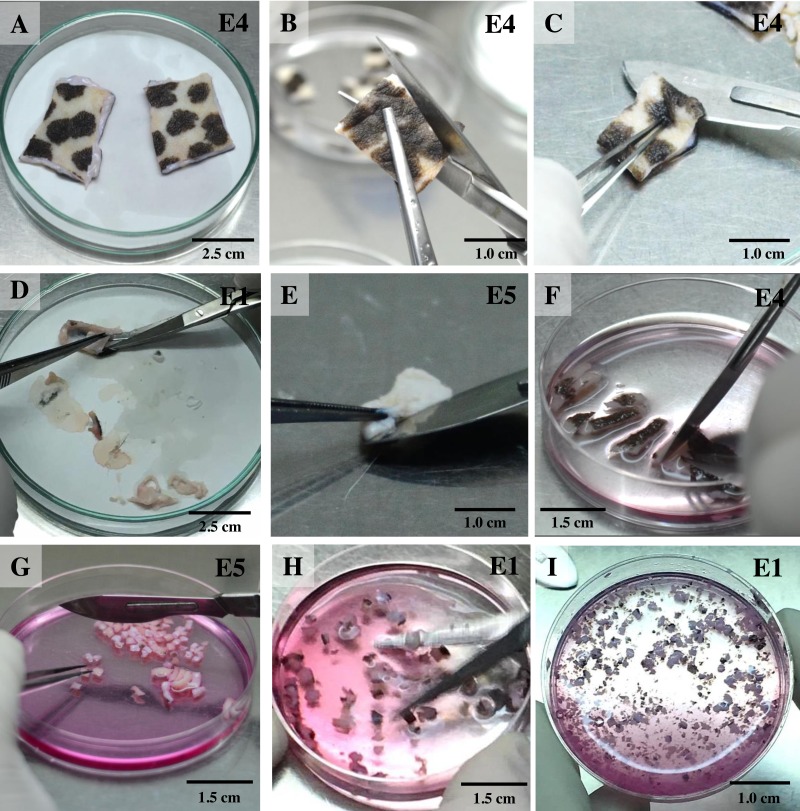
Ear explant preparation. (A) The ear skin samples were soaked in 70% ethanol, washed three times with washing PBS and dried on cellulose filter paper. (B) and (C) edges of the skin were trimmed, separating the front and back, respectively. (D) and (E) loose connective tissue and lobules of fat were removed and rinsed with washing PBS. (F) and (G) excised samples were cut with a scalpel into ∼1-cm-long strips, then chopped into pieces approximately 1 to 2 mm^2^ in size. (H) explants were washed with washing DMEM and then minced with iris scissors. (I) explants were digested in collagenase solution and incubated at 37 °C and 5% CO_2_ for 21 h; after that, the explant samples were further washed twice with PBS.

For the isolation of skin fibroblasts, ear tissues were prepared and cultured under a modified protocol, as described in previous reports (Mestre-Citrinovitz et al., 2016; Reiisi, Esmaeili & Shirazi, 2010; Rittié & Fisher, 2005; Vangipuram et al., 2013). In brief, the ear skin biopsies were dried on cellulose filter paper (Fig. 1A) (Whatman^®^; Sigma-Aldrich, St. Louis MO, USA), soaked once in 70% ethanol for 1 min, allowed to dry, and then washed three times with washing PBS containing ten times the routine antibiotic/antimycotic dose plus gentamicin. Tissue fragments were transferred into a 100 mm tissue culture dish using a sterile scalpel. A 5 mm perimeter was trimmed from the edges of the excised skin (Fig. 1B); the front and back of excised skin samples were then separated (Fig. 1C). Subcutaneous tissue (loose connective tissue and lobules of fat) was removed and rinsed with washing PBS (Figs. 1D and 1E). Excised samples were first cut with a scalpel into ∼1-cm-long strips, then chopped into pieces approximately 1 to 2 mm^2^ in size (explants) (Figs. 1F and 1G) and placed in washing DMEM containing ten times the routine antibiotic/antimycotic dose plus gentamicin. These skin explants were minced with iris scissors (Fig. 1H). Samples were centrifuged at 200 *g* for 10 min to remove the supernatant, then digested in collagenase solution (DMEM + 10% v/v collagenase type II + 10× antibiotic/antimycotic) in a 60-mm-diameter culture dish and incubated at 37 °C and 5% CO_2_ for 21 h (Fig. 1I). The next day, the explant samples were further washed twice with PBS. After the first 1 to 3 days of culture, explant samples were incubated in a 60-mm-diameter culture dish with explant medium containing 20% heat-inactivated FCS together with a routine antibiotic dose to protect against microbial contamination.

### Monitoring for outgrowth of primary cell cultures and contamination

**Figure 2:**
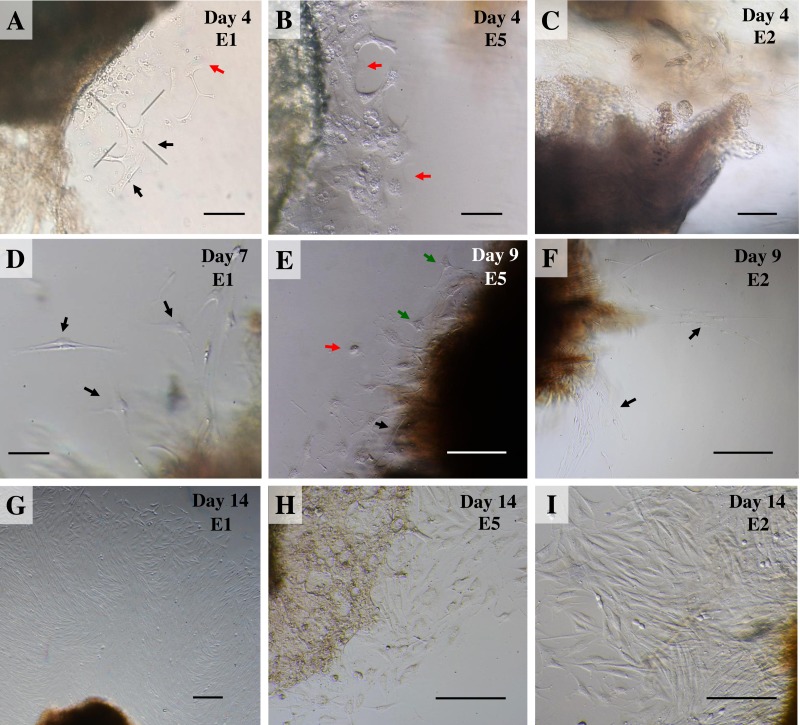
Outgrowth of primary fibroblasts and keratinocytes at P0 (passage zero) population from elephant skin explants. (A) and (B) exhibit an elephant skin explant with early (day 4) migration of keratinocytes (red arrows) and fibroblasts (black arrows) in explants from elephants E1 and E5, respectively. (C) illustrates an explant from E2 without any cell migration (at day 4) for comparison. (D) and (F) depict spindle-shaped fibroblasts which can clearly be seen at day 7 and day 9 around explants (black arrows) from elephants E1 and E2, respectively. (E) primary culture of skin cells, including keratinocytes (red arrow), melanocytes (green arrows) and fibroblasts (black arrow), from elephant E5 at day 9. (G–I) show the comparative confluence of primary outgrowing cells in explants from E1, E5 and E2, respectively, at day 14 of culture. Skin explants are dark shaded areas. All cultures were maintained in the same conditions, at 37 °C under 5% CO_2_. Scale bars = 200 µm.

**Figure 3:**
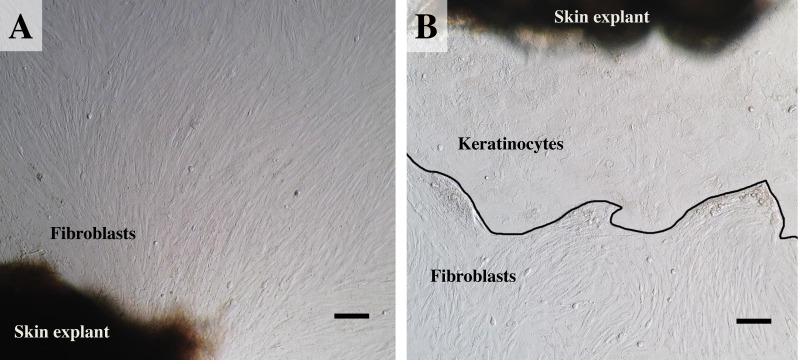
The labeled example illustrates the outgrowth of keratinocytes and fibroblasts from an elephant skin biopsy (E3) after three weeks of culture. (A) an example of fibroblast-like cells growing out of the elephant skin explant. (B) skin explant surrounded by polygonal cells, referred to as epithelial keratinocytes (squamous epithelia), that approach confluence until producing stratified multilayer sheets. Scale bars = 200 µm.

Once collagenase was removed, the cultures were monitored daily under an inverted microscope at low magnification to observe explant dislodging and the overall radial migration of primary cells around the explants (see Fig. 2), together with monitoring for any microbial or fungal contamination by checking the size, shape and movement pattern of the particles in the culture at high magnification. If microbial contamination was detected in any dish or flask, the entire contents were immediately discarded (Ryan, 2012). Initially, if no contamination was found at around day 4 of culture or if migration of primary cells was observed, the explants were moved from the dish to a culture flask. Tissue fragments were split into ∼20 smaller fragments in a T25 culture flask or ∼50–60 fragments in a T75 culture flask (making sure fragments did not touch each other). Tissue fragments were covered with a thin film of explant medium; culture continued until the primary outgrowing cells reached confluence around the explants or reached ∼80% confluence in culture flasks (Figs. 3A and 3B).

### Establishing secondary cultures

**Figure 4:**
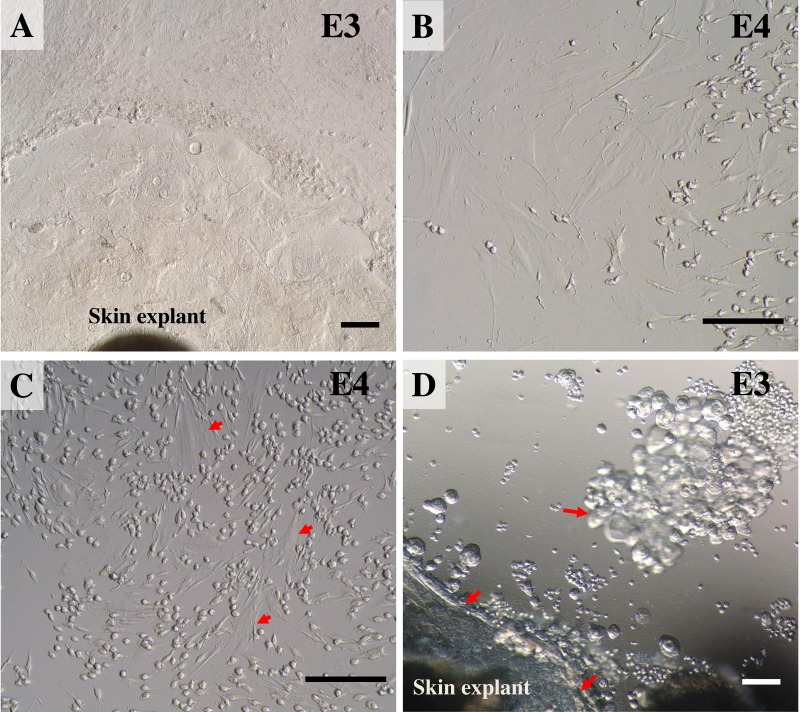
Establishing secondary cultures of fibroblasts. (A) the outgrowths surrounding the pieces of skin reached confluence and covered 80% of the flask. (B) 2 to 3 min after short trypsinization with 0.1% trypsin/EDTA application, fibroblasts were becoming rounded. (C) 3 min after short trypsinization, fibroblasts appeared rounded and were detached from the culture flasks, while keratinocytes were still attached to the plastic bottom. (D) after 15 min of trypsinization, both fibroblasts and keratinocytes were detached from the culture flasks. Scale bar = 20 mm.

Once reaching confluence (Fig. 4A), fibroblast cultures surrounding the pieces of skin were further expanded. The explant medium was poured out of the culture flasks and the cell surfaces washed with PBS three times. In these second and third passages, keratinocytes were removed from the cultures by short trypsinization with 0.1% trypsin/EDTA, as previously described (Hentzer & Kobayasi, 1975). The volume was adjusted as appropriate for different-sized vessels (for T75 culture flasks we added 1.0 mL of 0.1% trypsin/EDTA), followed by incubation at 37 °C. After a few minutes (2–3 min), when observed under a microscope, fibroblasts became rounded and began to detach from the plastic surface of the culture flask, due to the different attachment characteristics of keratinocytes and fibroblasts to the plastic culture surface, thus enabling separation of the two cell populations. To dislocate the remaining loosely attached cells, the flask was tilted to distribute the trypsin evenly over the culture surface. Separated fibroblasts were transferred to a sterile 15 mL centrifuge tube, followed by immediately adding at least five volumes of fresh growth medium containing 10% FBS and re-cultivating to expand cell numbers in a new T75 culture flask. Cultures were grown at 37 °C in a humidified atmosphere containing 5% CO_2_. During this time, keratinocytes were still attached to the plastic bottom and preserved their membranous shapes, as seen before trypsin treatment. To culture keratinocytes, cells were incubated at 37 °C for ∼10 min longer to disperse the cells; then the skin explants were removed and fresh growth medium added to re-cultivate keratinocytes. Moreover, to obtain a larger amount of fibroblasts, explants were left in the first flask for a second round of cell growth and incubated as before, while keratinocytes were separated and moved to a new T75 culture flask instead. The culture medium was changed once every 48 h. The 2nd to 4th passages of cultures of these cells were frozen for long-term storage stock. Fibroblasts at the 4th to 7th passages were used for further experiments.

### Routine trypsinization, cryopreservation and resuscitation of fibroblasts

Cryogenic preservation of elephant skin fibroblasts is generally the same as cryopreservation of most continuous cell lines: by using common dimethyl sulfoxide (DMSO), which permits long-term storage of cells in liquid nitrogen. After routine trypsinization of cells with 0.25% trypsin/EDTA and incubation for 5 min, cell suspensions were diluted with fresh growth medium and the sample thoroughly mixed. Cells were centrifuged at 200 *g* for 10 min. The pellets were re-suspended in freezing medium consisting of 50% DMEM, 10% DMSO and 40% FBS (v/v). Cell suspensions were aliquoted into cryogenic storage vials (at approximately 1.5–2.0 ×10^6^ cells/vial) and frozen at −80 °C in a deep freezer overnight. The next day the vials were transferred to a liquid nitrogen tank for long-term storage until used for further experiments.

To resuscitate fibroblasts or determine post-cryopreservation cell viability, the cells in the frozen vials were quickly thawed at 37 °C and promptly mixed with 6.0 mL of growth medium in a sterile centrifugal tube, then cultured in T75 culture flasks without centrifugation. Viable cells were counted manually using a hemocytometer and expressed as the % of live cells from the total cell count. The next day, attached cells were washed twice with PBS and new growth medium added.

### Measuring cell viability and generating a growth curve

Cells were counted to assess viability using a traditional cell-counting method, with a bright-line hemocytometer and trypan blue dye (Jauregui et al., 1981; Louis & Siegel, 2011). Briefly, cells were first trypsinized with a routine subculture. Cell suspension was mixed 1:1 with 0.4% trypan blue solution and let stand for 5 min at room temperature. Then 20 µL of the cell suspension was applied between the cover slip and the edge of the hemocytometer chamber and examined immediately under a microscope following the method of Louis & Siegel (2011). For measuring cell growth, seed cells at passage 6 (P6) of approximately 5,000 cells per well in 0.5 mL of growth medium in a 24-well plate were cultured under normal culture conditions. Viable cells in each well were counted over a 12-day period using a hemocytometer. The total count was calculated to obtain the growth curve and population-doubling time.

## Results and Discussion

### Tissue sample collection

To our knowledge this is the first report on the successful isolation and culture of skin fibroblasts from post-mortem ear tissues of a mature Asian elephant. In addition to providing convenience of tissue sampling, the ear serves as ideal tissue for fibroblast isolation, with an excellent number and quality of cells (Mestre-Citrinovitz et al., 2016). We collected ear samples after the elephant had died and the traditional requiem ritual had been performed by the mahout (Keyes, 1977). Including post-mortem examination (necropsy), ear cleaning and transportation time, the ear samples arrived at the lab around 10 to 24 h after the elephant’s death. This was still considered good quality remains for primary cell culture (Seluanov, Vaidya & Gorbunova, 2010). Previous studies have shown effective methods of preserving tissues and protocols to isolate outgrowth of fibroblast-like cells several days after death, taking into account different factors such as the storage temperature and animal species, e.g., 12 days after animal death from ears stored at 4 °C in goats and sheep (Silvestre, Sánchez & Gómez, 2004), 10 days post-mortem for sheep ear skin when skin tissues were exposed to around 25 to 26 °C (Singh & Ma, 2014), and recently published data showing a recovery method for fibroblast cells from goat skin up to 160 days post-mortem at 4 °C storage (Aoued & Singh, 2015). Of these protocols mentioned, the important point is that tissues were processed for good preservation within “an hour” of collection, which is almost impossible in the case of an elephant carcass in Thailand due to Thai cultural beliefs concerning the death ceremony. In this study, we failed to isolate any outgrowth of fibroblast cells from post-mortem tissues harvested 20 h after the animal’s death (elephant E6), even after culturing the explants for 1 month and finding no presence of contamination. It is possible that elephant E6 had been left in unsuitable conditions, i.e., at a high environmental temperature (∼33–35 °C in June) for too long a time, before ear samples were collected and transferred to the laboratory. Another important thing that should not be overlooked is the awareness of zoonoses. Wild animals may contain pathogens, such as anthrax tuberculosis and leptospirosis, that can be transmitted from animals to humans; and most samples were collected under field conditions, so taking care to avoid sharp surfaces that can pierce the skin and awareness of sterile technique are essential at all steps to minimizes any risks from infectious diseases (Kruse, Kirkemo & Handeland, 2004).

### Outgrowth of primary cell cultures

For our study we therefore adapted a collagenase digestion technique together with an explant technique to establish elephant fibroblasts (Mestre-Citrinovitz et al., 2016; Pruniéras, Delescluse & Regnier, 1976; Reiisi, Esmaeili & Shirazi, 2010; Rittié & Fisher, 2005; Vangipuram et al., 2013). Because trypsin digestion technique (Orazizadeh et al., 2015) was not completed to separate the dermis and epidermis layers in our study, outgrowth of epidermis (keratinocytes) and dermis (fibroblasts) could therefore be seen (Pruniéras, Delescluse & Regnier, 1976). Explant dislodging and overall radial migration around explants could be observed, as shown in Fig. 2 at low magnification. Both fibroblast-like and epithelial-like cells could be seen migrating from the tissue pieces 4–12 days after explanting. The earliest observed outgrowth around explants that dislodged was at day 4 of culture in E1 and E5 (Figs. 2A and 2B), compared with E2, E3 and E4 on the same day (Fig. 2C for comparison) in which primary cells were observed around day 7–10 (Figs. 2D–2F). We found outgrowths of epithelial keratinocytes around some explants in our experiments. They grew in squamous epithelia shapes until confluent and produced stratified multilayer sheets around the explants beginning after 4–7 days, while fibroblasts started later at around 7–10 days. At the time of fibroblast appearance during the following weeks the fibroblasts overgrew the keratinocytes and the cultures mainly consisted of fibroblasts with only small islands of epidermal cells around the explants (Fig. 3). Cells continued to proliferate and were subcultured when they reached 80% confluence (Figs. 3A and 3B).

### Establishing secondary cultures

During the first few minutes (2–3 min) of observation under a microscope, fibroblasts became rounded and began to dislodge from the plastic surface of the culture flasks, while the epidermal cells stuck to the bottom and preserved their membranous shapes (Figs. 4B and 4C). After 15 min of trypsinization, both fibroblasts and keratinocytes became detached from the culture flasks (Fig. 4D). After subculture, the fibroblast cells grew rapidly, gradually outgrowing.

**Figure 5:**
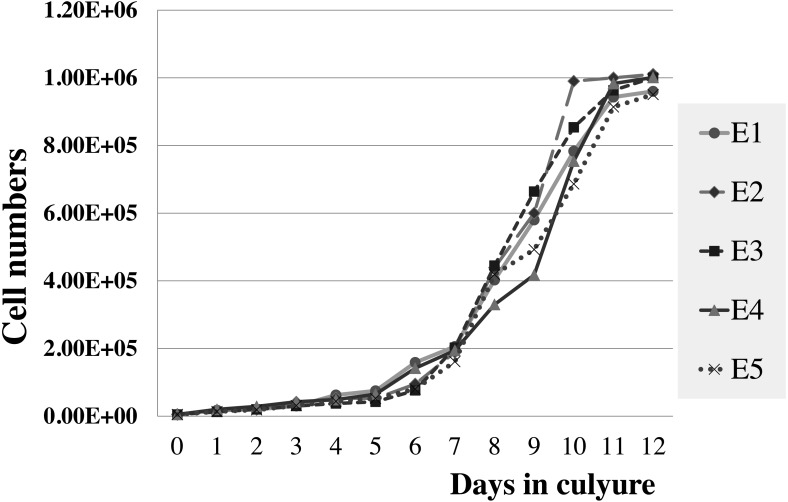
Growth curve of elephant skin fibroblasts at passage 6 (P6) obtained from five elephants.

### Measuring cell viability and generating a growth curve

The viabilities of elephant skin fibroblasts before freezing and recovery (percent cell viability was calculated immediately post-thaw) were around 97.3 ± 4.3%, and 95.5 ± 7.3%, respectively, when their post-freezing recovery and survival was improved with freezing medium consisting of 50% DMEM + 10% DMSO + 40% FCS (v/v). This indicates that the cells were grown in good culture conditions and that the freezing medium was appropriate. Note that at 10–20% FBS freezing medium the cells had very low recovery rates and cell morphology was different from cells observed prior to cryopreservation, which may be caused by apoptosis (Xu et al., 2010). In this study after 12 days continuous count the elephant skin fibroblast growth curve was as shown in Fig. 5. The curve was sigmoidal with a population doubling time (at the 6th passage) of about 25 h.

## Conclusions

This study has described the simple methods and requirements for isolating and culturing large numbers of primary cultures of *E. maximus* fibroblasts obtained from post-mortem ear skin tissues. The protocol presented here is a modified collagenase digestion procedure together with a simple explant procedure that can be applied to elephant fibroblasts. The problems we encountered and their successful resolution, employing the most efficient approach, are also demonstrated here. This knowledge should represent a step forward for the use of this technology for research in cell aging, in the treatment of age-associated impairments in dermal integrity and chronic wound healing, and in further cellular studies in Asian elephants. This study also suggests that suitable conditions during sample collection—such as post-mortem storage time, storage temperature, or other possible factors—may be associated with cell isolation success; however, a statistical test for this was not performed. Moreover, no correlation was observed between the age of an elephant at the time of death and the outgrowth of fibroblasts or the proliferation rate of cells. This should extend the usefulness of adult Asian elephant fibroblasts for further studies.

##  Supplemental Information

10.7717/peerj.4302/supp-1 Supplemental Information 1 Raw data for growth curve

## Additional Information and Declarations

### Data Availability

The following information was supplied regarding data availability: The raw data is included in a Supplemental File.
